# “We need to talk!” Barriers to GPs’ communication about the option of physician-assisted suicide and their ethical implications: results from a qualitative study

**DOI:** 10.1007/s11019-016-9744-z

**Published:** 2016-10-26

**Authors:** Ina C. Otte, Corinna Jung, Bernice Elger, Klaus Bally

**Affiliations:** 1 Institute for Biomedical Ethics, University of Basel, Bernoullistr. 28, 4056 Basel, Switzerland; 2 Institute of Primary Health Care, University of Basel, Klingelbergstr. 61, 4056 Basel, Switzerland

**Keywords:** Assisted dying, General practice, Communication, Medical ethics

## Abstract

GPs usually care for their patients for an extended period of time, therefore, requests to not only discontinue a patient’s treatment but to assist a patient in a suicide are likely to create intensely stressful situations for physicians. However, in order to ensure the best patient care possible, the competent communication about the option of physician assisted suicide (PAS) as well as the assessment of the origin and sincerity of the request are very important. This is especially true, since patients’ requests for PAS can also be an indicator for unmet needs or concerns. Twenty-three qualitative semi-structured interviews were conducted to in-depth explore this multifaceted, complex topic while enabling GPs to express possible difficulties when being asked for assistance. The analysis of the gathered data shows three main themes why GPs may find it difficult to professionally communicate about PAS: concerns for their own psychological well-being, conflicting personal values or their understanding of their professional role. In the discussion part of this paper we re-assess these different themes in order to ethically discuss and analyse how potential barriers to professional communication concerning PAS could be overcome.

## Introduction

When seeking assistance in dying, GPs are often a patient’s first point of contact (Meeussen et al. 2011; Sercu et al. 2012). GPs usually spend extended periods of time caring for their patients, and therefore often know their patients’ preferences and values very well (Meeussen et al. 2011; Sercu et al. 2012). Against this background, a patient’s request for the doctor to not only discontinue treatment, but also to assist him or her to die, is likely to create intensely stressful situations for both patients and physicians (van Marwijk et al. 2007; Georges et al. 2007). In our study and throughout this manuscript we will refer to the act of a physician assisting a patient in dying as physician-assisted suicide (PAS). We define physician-assisted suicide as “a physician providing a prescription of a sufficient dose of drugs to enable a patient with a terminal illness to kill him-or herself”.

Earlier studies have shown that patients and their relatives highly value the opportunity to talk to the treating physician about the option of PAS: patients state that discussing the option of PAS as a possible “way out” helps them to deal with their situation and can help them to relieve stress (Rabow and Markowitz 2002; Johansen et al. 2005; Back et al. 2002). Furthermore, patients’ requests for PAS can also be an indicator of unmet needs or concerns of patients (Bascom and Tolle 2002). Therefore, several factors are very important in order to ensure the best possible patient care. These include competent communication about PAS as well as the assessment of its origin, the sincerity of the patients’ wish to die, and other viable alternative treatment options (Back et al. 2002; Gastmans et al. 2004).

From a legal perspective, PAS in Switzerland is not explicitly permitted by legislation; however, assisting in a suicide has not been a prosecutable act for almost a century (Bosshard et al. 2002). This is provided that the person seeking assistance is competent and the assister is not motivated by self-interest, pursuant to Article 115 of the Swiss Penal Code (Cassani 1997). However, even though PAS was not illegal, the code of professional conduct originally did not support the participation of physicians (Hurst and Mauron 2003). This changed in 2004, when the Swiss Academy of Medical Sciences (SAMS 2004) published its medical-ethical guidelines on PAS, which state that “it is not part of a physician’s activities because it is contrary to the goals of medicine, but it may be considered by the physician if the person requesting it fulfils certain criteria: is within days or weeks of the end of life, is competent and the wish is well considered and not due to external pressure, and alternative means of assistance have been discussed” (SAMS 2004).

It is therefore the task of GPs receiving requests to establish whether a patient fulfils the listed criteria; this requires the competent and professional handling of this issue (Back et al. 2002). Thus, it is essential to explore how GPs communicate about PAS when receiving requests from patients in practice (Back et al. 2002). Understanding their reactions and experiences of PAS queries, and the rationales behind their responses to such requests, is important in order to fully understand any potential shortcomings, barriers or psychological discomfort associated with this issue. However, the available literature is often limited to studies detailing physicians’, patients’, and other stakeholders’ attitudes towards AS in general (e.g. Hussain and White 2009; Nordstrand et al. 2013) and arguments pro and against its legalization (e.g. Lee et al. 2009a, b; Robinson and Scott 2012; Rurup et al. 2005; Wolfe et al. 1999). Furthermore, the main approach presented is often aimed at the elaboration of GPs’ attitudes towards PAS in general, but not upon their way of actually communicating or responding to requests for AS (e.g. Craig et al. 2006; Meier et al. 1998).

In this study, we chose a qualitative research method (semi-structured interviews) in order to explore in depth this multifaceted, complex topic while enabling general practitioners (GPs) to express possible difficulties they experience when asked to communicate about this matter. The gathered data gave us insight into potential barriers to professional communication about PAS while also giving us rich data with which to ethically analyse GPs communication in practice.

## Methods

This paper describes results from a Switzerland-wide study entitled “Conditions and Quality of End-of-Life Care in Switzerland—the Role of General Practitioners” which was funded by the Swiss National Science Foundation. The aim of this study is to conduct a detailed exploration of the attitudes and difficulties of GPs who administer palliative care in primary practice. Therefore, the study design included a qualitative research part at the beginning of the research project, which is particularly suited to understanding GPs attitudes, values and difficulties when it comes to palliative care and requests for PAS (Pope and Mays 1995). As one of the two steps (focus groups and semi-structured interviews) in the qualitative section of the study, 23 qualitative interviews with general practitioners were conducted and analysed.

### Sampling and data collection

A purposive sampling of 30 GPs was chosen from the FMH (Swiss Medical Association) list in order to obtain the maximum variety in terms of practice size (group vs. single), location (practices in different cantons and in urban, rural or suburban regions), and doctors’ gender and age. Selected GPs were contacted via an e-mail outlining the research. Semi-structured face-to-face interviews, approximately 1 h in length, were conducted with the participants. These participants were based in the French, Italian, and German speaking areas of Switzerland. The interviews took place between December 2012 and February 2013. The interviews were conducted by IO and CJ (both authors of this paper). Both interviewers (IO and CJ) are sociologists specializing in qualitative research methods and interviewing techniques. An interview guide was used for all interviews, which evolved as new insights were gained during the data gathering process and led to a more in-depth exploration of this topic. Among the question sets concerning administering palliative care and their networking with other institutions and stakeholders, the participating GPs were asked about their reactions to and handling of requests for assisted suicide. The study was approved by the competent ethics committee (Ethics Committee northwest/central Switzerland “EKNZ”) in November 2012 and all participants provided informed consent.

### Analysis

The interviews were transcribed verbatim (using the transcription software “F4”). IO and CJ carried out an independent analysis of all transcripts (using Atlas.ti). Additionally, a secondary coding was performed by KB and BE. Critical reviews of each analysis of each interview were performed in order to help us to become aware of our own backgrounds and potential bias (reflexivity) (Malterud 2001; Malterud 2002). The codings were then reviewed by two independent researchers to ensure inter-rater reliability.

The coders followed Mayring’s steps of content analysis (Mayring 2003; Lamnek 2010). In a first step, the data was coded separately by IO and CJ, moving from concrete passages to more abstract levels of coding including emerging themes. Both coders then discussed their codes and re-coded the data again. After five interviews a preliminary coding guide was developed which was adapted continuously throughout the analysis, adding new codes emerging from the material, if necessary. In team meetings all findings were critically tested and discussed by all coders. Any disagreements were solved by discussion. Since the coding system remained the same for the final interviews and no new codes/themes emerged, we concluded that we had reached saturation.

## Results

Of the 23 GPs who participated in this study, three interviewees declined to answer questions about PAS due to personal discomfort. Of the remaining 20 GPs, about two-thirds of the interviewees clearly stated that they would not assist with a patient’s wish to proceed with PAS. A few of these interviewees also reported that they discourage patient requests in advance by saying that performing physician assisted suicide is not an option for them. The remainder of the GPs were either supportive of or indifferent to PAS. Those GPs who support PAS stated that they believe it is a compassionate response to a medical need and prescribed the needed medication. Some of them reinforced their position with the rationale that it is good for patients to know about a possible way to end their suffering.

Participants in our study received one to three requests for PAS in their career. The GPs who had chosen to refuse to assist a patient’s suicide comprised the largest group in the study and provided the most insight into their handling of requests for PAS. As such, and because we were particularly interested in possible barriers to their patient communication about PAS, this paper mainly focuses on the analysis of their reasons and arguments. We identified three main themes concerning how GPs accounted for their stated refusals to assist a patient’s suicide:

### Theme 1: Handling of emotional and psychological impact

GPs who stated that they avoid talking about PAS requests emphasized their uncertainty about their ability to cope emotionally with assisting a patient in ending their life. They stated that they fear their own psychological health might be at risk. Instead of PAS, they try to find a way to support the patient without intentionally causing death, for example by giving the patient morphine. Especially in cases where they have a long and well developed patient–physician relationship, the emotional impact of PAS requests increases due to their personal connection to the patient. While they could empathize with terminally ill patients’ wishes to die, the feeling of not being able to handle the emotional, ethical, or psychological impact was overwhelming for them. A few physicians reported that they always felt relieved when they did not have to talk about a request for PAS.

GP4: I do not feel competent to deal with the topic of assisted suicide, and I do not want to either. Especially for my personal psychological health, when I know the patient for a while. I find this legitimate, and I am always relieved when I do not need to think about this topic.

GP19: One of the things I try to tell them is that I cannot bear the idea of killing one of my patients. I’m not strong enough for that, I cannot cope with it.

GP23: When someone asks for help, I explain that I do not do assisted suicide. It’s me, I cannot do it, I feel like I would not be capable—psychologically. So I admit I would never do it. I would give them morphine or something like that.

GP5: To me, it is important to clarify the situation as early as possible. I tell them that I could never do it. I say that at the very beginning. This way I can avoid discussing this topic. I guess one consequence of my behaviour is that I choose the easiest patients to treat.

### Theme 2: Religious beliefs and moral values

Some of the interviewed GPs who did not want to assist a suicide commented that their opinions are related to their personal values. However, all of them acknowledged that situations may arise in which a request for PAS is quite understandable. While these GPs acknowledge and respect the wishes of their patients, they stated that their own “set of moral values” was the reason for their refusal to assist their patients in ending their lives. They feel committed to relieving a patient’s suffering and most agree that physician assisted suicide might, in some cases, be an option for patients. However, they do not want to take an active role in PAS. They would rather search for other options for their patients, such as improved palliative care, the transferal to another doctor or psychological support. As shown in the following quote, the refusal to assist a patient in dying can also lead to a postponement of the conversation about this option to a later moment “when the time has come”.

GP17: Some patients requested it, but I told them: “No, do not rely on me to give you the prescription of this product, no”. I tell them clearly: “do not count on me, it (PAS) is against my beliefs, but I respect your choice, and I’m ready to help you and to accompany you otherwise. We also do not have to talk about this topic now, we will see when the time has come”.

However, as shown in the following quotes, though most participants acknowledge that situations may arise in which a request for PAS is understandable, they still cannot participate in this procedure due to their personal values and beliefs:

GP18: I can totally understand why an assisted suicide can be meaningful. I just cannot support it. It simply challenges my beliefs.

Some of them also think that there are other, sufficient options such as palliative care or psychological support: GP13: I had to tell that patient that I am sorry, but that it is not compatible with my own philosophy and that there are other sufficient options such as palliative care or psychological support.

### Theme 3: Conflicts with professional role

Several GPs contend that PAS is not a part of their professional role. They believe their duties are to ensure the patients’ quality of life, to alleviate pain and suffering, and to provide support to patients and their families. They also fear that their involvement with PAS could lead to confusion about their roles as doctors or could make assisted suicide look like a “normal medical procedure”. Some of them underlined that patients’ wishes for an assisted suicide were a psychological issue and possibly avoidable. As soon as the patients received treatment, either from a psychiatrist for their psychological suffering or from a GP who is able to relieve their physical pain, the wish for an assisted suicide, in their opinion, vanishes.

GP23: Me, I listen, listen to my patients, but I cannot give them the medication, I cannot see myself doing it. I think it’s not my role. Another aspect is…, it seems clear to me, that we cannot declare assisted suicide as a normal medical procedure, because otherwise the pressure of our society on aged people might grow to that extent that those people in retirement homes might get the feeling that it is their duty to commit assisted suicide, because they only cost money or because they are “useless”.

GP20: It’s quite unusual but when it happens it often demonstrates that they (the patients) feel weakened, they are now in a situation where they feel worse. If you manage to read behind the suffering and if you manage to answer to this pain most people forget they talked about suicide because they have their answer.

GP15: I don’t talk about it because it is not part of my job, the person needs care I cannot give in my cabinet. Generally, I try to convince the person to see a psychiatrist.

GP12: I think if I was promoting suicide, a lot more of my patients would do so. We are really underestimating the influence we have as doctors, especially during end of life care, where people need to give up more responsibilities about themselves. So for me, assisted suicide can never be a part of my professional role.

## Discussion

As shown in the introduction, competent communication about PAS is important on different levels: e.g. a patient’s request for PAS can be an indicator for unmet medical needs. Furthermore, talking about AS can give patients the feeling of regaining control about their life and is therefore a possible means of relieving stress in patients (Rabow and Markowitz 2002; Johansen et al. 2005; Bascom and Tolle 2002; Back et al. 2002).

Participants in our study received one to three requests for PAS in their career, which is reflective of the national average of assisted suicide requests experienced by GPs in Switzerland (Brauer et al. 2014). During the interviews for this study, GPs illustrated possible barriers faced when confronted with requests for PAS; they have to weigh the suffering of a terminally ill patient on the one hand against their own psychological well-being, personal values and understanding of their professional role on the other.

In this section we re-assess these different themes that emerged from the interviews in order to ethically analyse how potential barriers to this kind of communication could be overcome.

### Theme 1: Expected psychological impact

GPs who addressed this theme based their rejection mainly on their feeling of not being able to handle the emotional impact of PAS. In order to better understand their feelings, it might be important to acknowledge that although it is legally possible, the handling of requests and the procedure of PAS is still relatively new to GPs. Before 2004, professional guidelines in Switzerland had considered PAS to be incompatible with the aims of medical practice (SAMS 1995). Thereafter, the guidelines of the Swiss Academy of Medical Sciences regarding PAS were broadened (SAMS 2004). This rather recent and still controversial change may therefore leave GPs feeling insecure about the process of PAS, its legal prerequisites and how to proceed professionally.

As a step towards overcoming this barrier, other studies have shown that further education and training can have positive effects on GPs and their capability to deal with the potential psychological impact of PAS (Gastmans et al. 2004; Gamondi et al. 2013a). Therefore, the topic of PAS and its possible effects on medical professionals should be included in the official vocational training program for general practice. Currently, postgraduate training in Switzerland seldom covers the subject of PAS (Eychmüller et al. 2015). Additional knowledge concerning PAS and its legal and ethical prerequisites could help to limit the feeling of discomfort expressed by GPs.

Concerning GPs’ concerns about the expected psychological impact, van Marwijk et al. (2007) have shown that ensuring that sufficient time is available for all involved parties to deal with the emotional component of PAS could be another way to reduce potential discomfort. Other studies have shown that team consultations and guided group supervisions of all medical professionals involved, paired with a strengthened education, can help GPs to handle the emotional and psychological impact (Berghe et al. 2013). Berghe et al. (2013) further report that medical professionals who formerly declined patients’ requests for similar reasons found it helpful to accompany a patient undergoing the procedure as a witness, learning that the patient was relieved to gain back control and grateful that his “final days did not have to last any longer”, whether or not all medical possibilities had been exhausted. As a result, these medical professionals were convinced that the procedure could be part of “genuinely good care” which then minimized their discomfort (Berghe et al. 2013).

### Theme 2: Religious beliefs and moral values

Interviewees in this group found PAS to be in direct conflict with their own morals and values. They base their refusal to communicate with their patients about PAS on personal ethics. They are not against PAS in the context of their profession, but because of their individual opinions. The GPs in our study were aware of their personal struggle with this topic and acknowledged that medical professionalism requires them to be aware that their own personal values might have an impact on offered treatment choices and therefore on patient autonomy.

One possible impact on patient autonomy was apparent when GPs reported their attempt to postpone final decisions with their patients by offering to accompany them and to talk about PAS “when the time has come”. However, by encouraging the postponing of a final decision, GPs risk compromising patients’ autonomy since patients need to fulfil certain criteria for PAS, such as displaying competence and the ability to take the PAS medication by themselves (Guillod and Schmidt 2005). Rather than postponing the decision, a better option to ensure patient autonomy could be the transfer of the patient to a colleague or a right-to-die organisation once a patient’s concrete decision to proceed with assisted suicide has been made. However, in terms of continuity of care, the option of referring a patient to a different colleague or organisation should be thoroughly planned and well considered, since the patient will then be treated outside his or her familiar care environment, which might not be optimal (Berghe et al. 2013).

Nevertheless, it is also important to note that despite all the support that can be offered (e.g. better education on the subject, group consultations, guided team supervision, accompanying of a patient undergoing PAS as a witness etc.), it must be understood that no-one can be compelled to participate in any form of suicide assistance if it is incompatible with their own moral stance or endangers their psychological health (Ersek 2004). This dilemma requires a personal decision of conscience and as such must be respected as long as it does not prevent a GP from offering other options to the patient (e.g. a transfer to a different GP) in order to ensure full patient autonomy.

### Theme 3: Professional role

Some interviewees stated that their main reason to reject a conversation about PAS is because they see conflicts with their professional role when being asked for their assistance. Their understanding of the medical ethos, with the aim of healing patients while trying to avoid causing additional harm, may contribute to their way of handling PAS requests. However, some of the GPs acknowledged that there are additional medical goals that physicians have to take into account, e.g. the respecting of patients’ personal values and priorities. The Swiss Academy of Medical Sciences has also identified this dilemma. According to their guidelines, PAS cannot be part of a doctor’s role because it contradicts the aims of medicine (SAMS 2004). In paragraph 4.1 they state that the proper task of doctors is to relieve patients’ suffering, not to offer them assistance in committing suicide (SAMS 2004). However, one could argue that refusing to relieve a patient’s suffering by providing PAS amounts to causing harm by omission. Further, the consideration of a patient’s wishes is fundamental for a good doctor–patient relationship. Following Andorno (2013), the argument against doctor’s involvement in their patients’ suicide is based on the risk of creating confusion about the proper aim of the medical profession (Andorno 2013). Some of the participants’ responses in this study alluded to the same reasoning: they fear assisted suicide could become a “common and frequent” procedure once they signal their acceptance. According to Martin et al. (2011), focusing on healing as the main aim of medicine is potentially ambiguous as this term does not completely subsume medical practice. There are many medical practices that are clearly not specifically healing in nature, but that are still regarded as ethically acceptable and compatible with the medical ethos. Martin et al. (2009) state that these measures are accepted because “providing care in accordance with the personal goals and values of the patient is an additional goal of medical practice besides healing”. Furthermore, the original definition of the aim of healing might be outdated and not fully apply to newer developments in aging societies (e.g. long term diseases where only palliative and not necessarily curative treatments are possible) (Martin et al. 2011).

Some of the participants stated that PAS would, in their opinion, not be requested if better palliation of pain and/or methods of decreasing psychological distress were made available. From the literature, it is known that there are three major factors in suffering at the end of life: pain and other physical symptoms, psychological distress, and existential distress (described as the experience of life without meaning) (Foley 1997). While there is some progress in undergraduate teaching on palliative care, as noted above, postgraduate training in Switzerland seldom covers the topic of PAS. Furthermore, Swiss data from 2008 suggests that physicians are inadequately trained in assessing and managing the multifactorial symptoms commonly associated with patients’ requests for PAS (Pereira et al. 2008). Training in palliative care is an obligatory part of the learning objectives in medical schools but only a few Swiss universities currently offer formal courses (Eychmüller et al. 2015). The average number of mandatory hours of palliative care education is 10.2 h, which falls significantly short of the 40 h recommended by the European Palliative Care Association’s Education Expert Group (Pereira et al. 2008; Eychmüller et al. 2015). An increase in the number of mandatory hours of palliative care education (ideally also covering the topic of PAS) could help to prepare GPs for handling of requests as well as supporting them when it comes to assessing in depth the origin of a patient’s wish for PAS. This is especially apparent when it is taken into consideration that other studies have shown that this training has positive effects (Gastmans et al. 2004). Perhaps even more so, considering studies have shown that patients’ decisions are highly influenced by the actual and perceived belief of pain relief (Foley 1991; Linton and Shaw 2011). Their consideration of requests for PAS was found to be directly linked with physical or psychological symptoms (Haverkate et al. 2001) which were also mentioned by some GPS in this study. It is therefore of utmost importance to handle requests professionally and to evaluate whether an assisted suicide request is made because of suffering that can possibly be alleviated through other methods. However, it is also important to note that more and more patients request PAS not only because of physical or psychological symptoms but because they fear losing their autonomy (Gamondi et al. 2013b; Fischer et al. 2009).

## Conclusions

Our qualitative study has shown that patients’ requests for PAS can create stressful situations for GPs. Participants who reject requests for PAS stated to either feel that (a) they are not able to handle the emotional impact of PAS, (b) PAS to be in direct conflict with their own values or (c) that their assistance would contradict their understanding of the medical profession. Some of the participants also reported to avoid conversations about this topic even though it would be important to assess the origin of a patient’s wish for PAS. A possible approach to improve the situation could be the involvement of Swiss right-to-die organisations such as EXIT. That way, GPs would not necessarily be required to participate in the actual procedure of PAS which could partly minimize the discomfort associated with the topic. However, in order to elaborate to what extent this approach could be useful, further research is required.

Furthermore, an increase in the number of mandatory hours of palliative care education (also covering the topic of PAS) could help to prepare GPs for the handling of requests as well as supporting them when it comes to assessing in depth the origin of a patient’s wish for PAS. This could also help physicians in cases in which patients’ values differ from their own to have a professional conversation about the topic of PAS. Even in cases of disagreement, the willingness of the treating GP to talk about the option of PAS was shown to be very meaningful, not only to patients but also their families.

### Strengths and limitations

A key strength of this study is its use of a qualitative method to explore a multifaceted topic, enabling GPs to express their own attitudes towards PAS. However, the study sample may not have represented the full range of GPs’ views on the topic, since it was not specifically chosen to explore the issue of PAS. Other selection biases due to the recruitment process are possible as the study was announced under the title of “conditions and quality of end-of-life care in Switzerland—the role of general practitioners”. This announcement could result in a bias towards the participation of physicians who feel confident regarding palliative care and/or advance care planning.

While the topic of PAS is particularly sensitive, and may have legal implications, we recognize that GPs may prefer to avoid portraying a positive attitude towards PAS (social desirability). However, anonymity and congruency with other studies along with additional received statements that are not necessarily socially desirable (e.g. their general positive attitude towards PAS) lead us to conclude that this bias remains small. In order to gain better insight into communication about PAS in practice, we also considered the method of participant observation instead of interviews, as well as a combination of both methods. However, since requests for PAS are very infrequent and rather rare (the participating GPs received one to three requests for PAS during their career which is reflective of the national average), an observation of the conversation between GPs and their patients about PAS was not a feasible option due to the very limited timeframe of this study.
